# Synthesis and crystal structure of [1,3-bis­(2,6-diiso­propyl­phen­yl)imidazol-2-yl­idene](iso­cyanato-κ*N*)gold(I)

**DOI:** 10.1107/S205698902400046X

**Published:** 2024-01-19

**Authors:** Abolghasem Bakhoda

**Affiliations:** a 1Department of Chemistry Towson University 8000 York Road Towson, MD 21252, USA; University of Durham, United Kingdom

**Keywords:** crystal structure, gold(I), iso­cyanate, *N*-heterocyclic carbene

## Abstract

The title complex, [Au(NCO)(C_27_H_36_N_2_)], was synthesized by ligand metathesis from [1,3-bis­(2,6-diiso­propyl­phen­yl)imidazol-2-yl­idene]gold(I) chloride and sodium cyanate in anhydrous tetra­hydro­furan and crystallized from toluene at 233 K in the ortho­rhom­bic space group *P*2_1_2_1_2_1_, as a neutral complex with the central Au atom di-coordinated by an *N*-heterocyclic carbene [Au—C = 1.963 (2) Å] and an iso­cyanate [Au—N 1.999 (2) Å] ligands, with a linear CAuNCO moiety. The crystal packing is consolidated by C—H⋯O hydrogen bonds.

## Abstract 2

The title complex was synthesized by ligand metathesis from [1,3-bis­(2,6-diiso­propyl­phen­yl)imidazol-2-yl­idene]gold(I) chloride and sodium cyanate in anhydrous tetra­hydro­furan and crystallized from toluene at 233 K as a neutral complex with the central Au atom di-coordinated by an N-heterocyclic carbene and an iso­cyanate, with a linear CAuNCO moiety.

## 1. Chemical context

Transition-metal complexes with N-heterocyclic carbene (NHC) ligands have been frequently used as ligands in inorganic and organometallic synthesis, chemical catalysis, and medicinal chemistry (Hopkinson *et al.* 2014 ▸; Collado *et al.*, 2021 ▸). NHC complexes of gold are typically linear dicoord­inate Au^I^ complexes, however, square-planar Au^III^ complexes are also known (Baron *et al.*, 2017 ▸). The former, where the dicoordinate state of Au^I^ is sterically and electronically stabilized by NHC ligands, have inter­esting bonding properties (Pyykkö, 2004 ▸) and are prospective as catalysts (Collado *et al.*, 2021 ▸) and medicines (Dada *et al.*, 2017 ▸). An important class of Au^I^ compounds are those with pseudohalide anions, such as CN^−^, SCN^−^, N_3_^−^ or NCO^−^. In the present work, we attempted to synthesize an Au^I^–cyanato complex, (IPr)AuOCN, where IPr = 1,3-bis­(2,6-di-iso-propyl­phen­yl)imidazol-2-yl­idene, as no Au^I^–cyanato complex had been isolated and structurally characterized previously, while those of Cu and Ag are very rare (see Section 4). In the attempt, we reacted (IPr)AuCl with sodium cyanate in anhydrous THF, which yielded the title *iso*cyanato complex (IPr)Au—N=C=O (**1**), as proven by X-ray crystallography. 
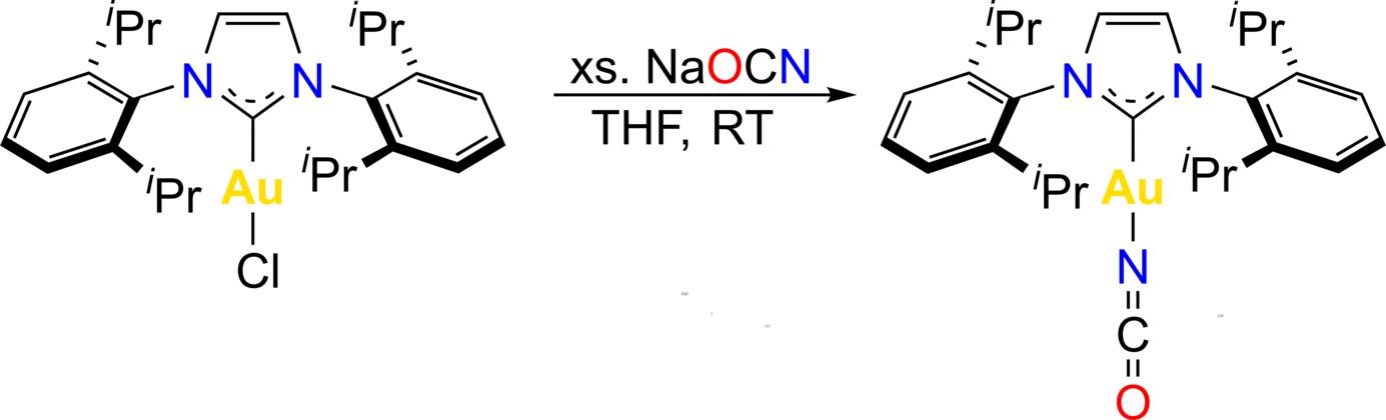


## 2. Structural commentary

**Figure 1:**
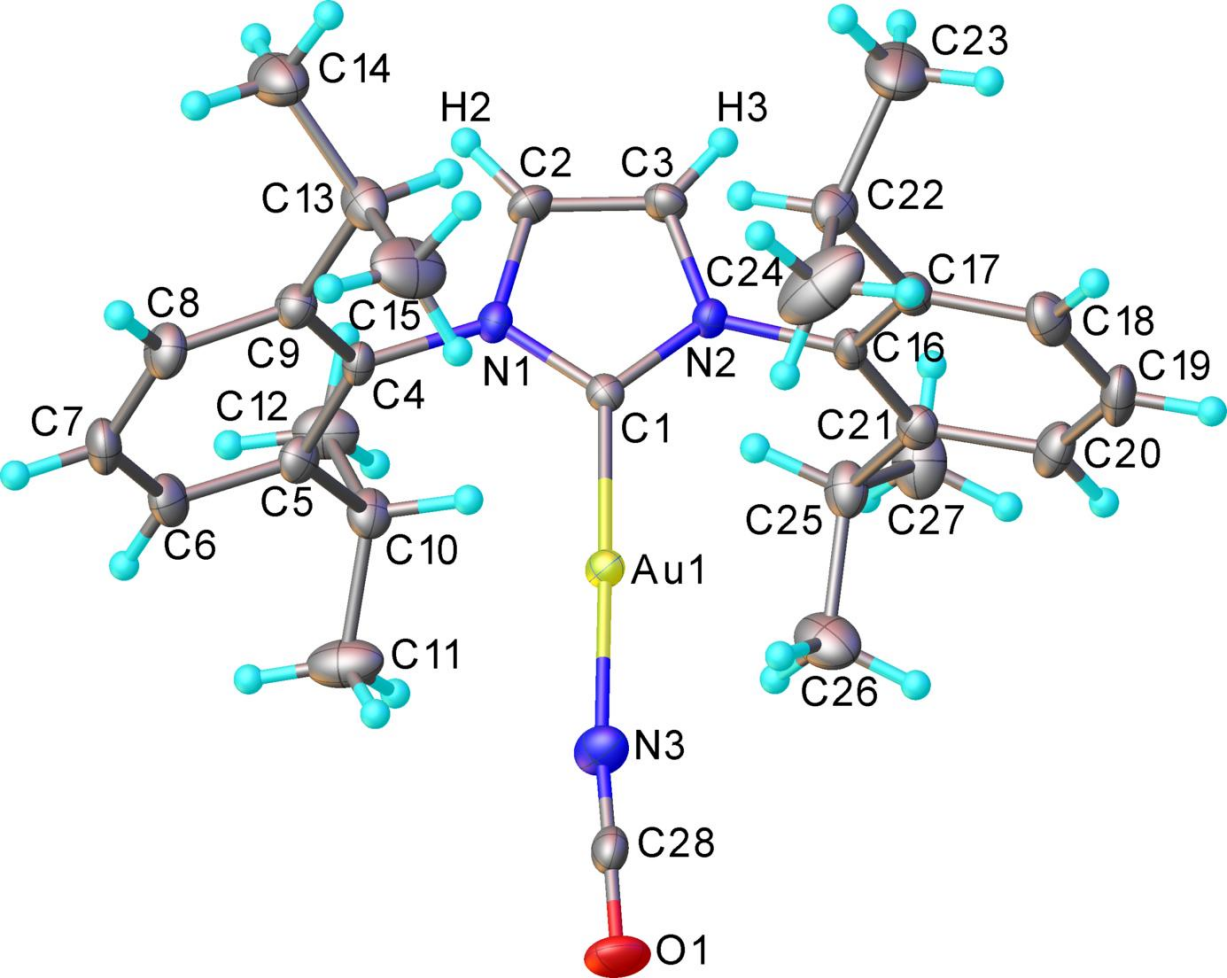
The mol­ecular structure of (IPr)Au—N=C=O (**1**), showing atomic displacement ellipsoids at 50% probability.

Crystallographic results (Fig. 1 ▸) unambiguously show the presence of an iso­cyanate (rather than cyanate) ligand that is N-bonded to the Au atom, with a nearly linear Au1—N3—C28 angle of 173.8 (2)° and the bond lengths N3—C28 [1.130 (3) Å] and C28—O1 [1.210 (3) Å] in the normal ranges found for metal–iso­cyanates (see Section 4), of 1.11–1.15 and 1.18–1.23 Å, respectively. The Au atom coordination is also linear [C1—Au—N3 178.14 (11)°], the Au—N3 and Au—C1 bond lengths of 1.999 (2) and 1.963 (2) Å, respectively, are not unusual for iso­cyanate and carbene ligands in previously reported Au^I^ complexes (listed in Section 4).

The IR spectrum of **1** (ATR, Thermo Scientific Nicolet iS10 spectrometer) shows the asymmetric stretching frequency ν_NCO_ of 2234 cm^−1^, in good agreement with other iso­cyanate Au^I^ complexes (see Section 4).

## 3. Supra­molecular features

**Figure 2:**
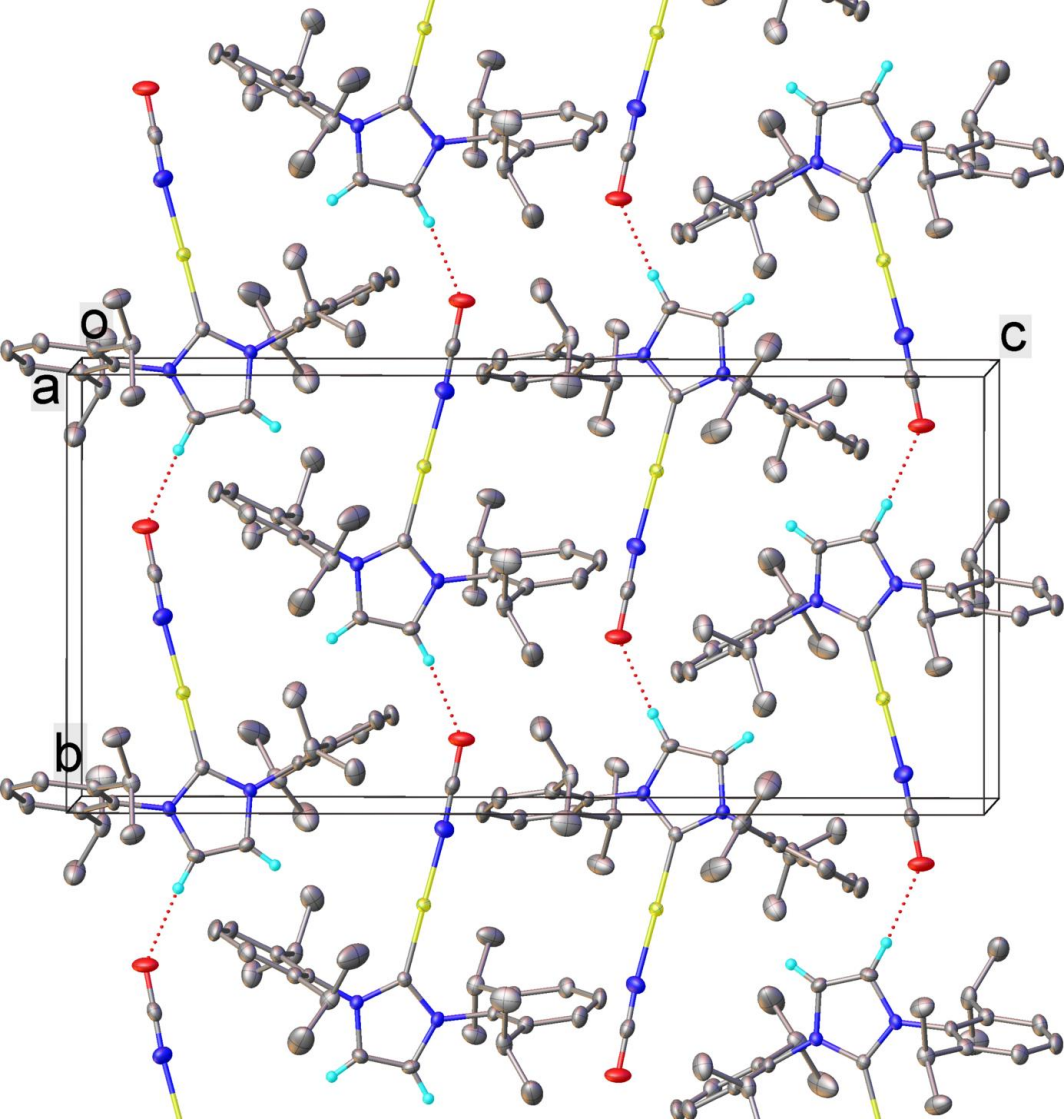
Crystal packing of **1** with hydrogen bonds shown as red dotted lines. Au atoms are shown in yellow, N in blue and O in red. H atoms except H2 and H3 are omitted for clarity.

In the crystal, discrete mol­ecules of **1** are oriented with their CAuNCO ‘rods’ roughly parallel to the crystallographic *b* axis, with no indication of π–π stacking. While di-coordinate Au^I^ atoms (*d* ^10^ centers) often form attractive aurophilic Au⋯Au inter­actions, which play an important role in determining the solid-state structures of Au^I^ complexes (Pyykkö, 1997 ▸), in the structure of **1** no such inter­actions occur, the closest Au⋯Au distance being 7.738 Å. This is probably due to effective shielding of the Au center by 2,6-di-iso-propyl­phenyl groups. The inter­molecular hydrogen bond C2—H2⋯O1(*x*, *y* + 1, *z*) is relatively strong, with the distances C⋯O 3.127 (3), C—H 0.94 (3), H⋯O 2.25 (3) Å and C—H⋯O angle of 155 (2)° (Fig. 2 ▸). The asymmetric unit of **1** contains only one mol­ecule.

## 4. Database survey

Structurally characterized cyanate complexes of Group 11 metals with *M*—O—C≡N core (*M* = Cu, Ag, Au) are very rare. In the literature, there are only six examples of copper cyanato complexes and only one example of a silver cyanato complex is known so far (CSD version 5.43, last update November 2023; Groom *et al.*, 2016 ▸). Thus far, there is no example of an isolated and structurally characterized Au^I^–cyanato complex in the literature.

A search of the CSD (version 5.43, last update November 2023; Groom *et al.*, 2016 ▸) using CONQUEST (Bruno *et al.*, 2002 ▸) revealed four Au^I^–iso­cyanate coordination compounds, *viz*. (Ph_3_P)AuNCO (CSD refcode DUCRIC, Bosman *et al.* 1986 ▸), two complexes with the composition *L*AuNCO, where *L* is an NHC ligand, *viz*. 1,3-di-*tert*-butyl­imidazol-2-yl­idene or 1,3-dibenzyl-4,5-diphenyl-2,3-di­hydro-1*H*-imidazol-2-yl­idene (FAWZOT, Baker *et al.*, 2005 ▸; LAMLIX, Dada *et al.*, 2017 ▸), as well as one complex of the composition *L*AuNCO, where *L* is a cyclic(alk­yl)(amino)­carbene (QANMUQ; Romanov *et al.*, 2017 ▸). The IR spectra of these show the characteristic ν_NCO_ bands at 2204, 2232, 2243 and 2229 cm^−1^, respectively.

## 5. Synthesis and crystallization

An aluminum-wrapped oven-dried 25-ml Schlenk flask was equipped with a stirring bar and charged with IPrAuCl, purchased from Strem (100 mg, 0.16 mmol) and sodium cyanate (52.6 mg, 0.81 mmol) under an anhydrous di­nitro­gen atmosphere inside a glovebox. Anhydrous THF (15 ml) was added, and the resulting suspension was stirred overnight at room temperature. The solvent was removed, the residue dissolved in anhydrous toluene and filtered through short pad of silica (1.5 cm). This filtration procedure proved crucial for the efficient removal of small amounts of impurities and increased the stability of the product. The colorless filtrate was concentrated and hexane was added to precipitate complex **1**, the solvents were deca­nted off and the residue dried *in vacuo*. Yield: 36 mg, 36%. The product was recrystallized from a concentrated toluene solution at 233 K inside a di­nitro­gen-filled glovebox. ^1^H NMR (400 MHz, CDCl_3_): *δ* 7.51 (*t*, *J* = 8 Hz, 2H, C*H* aromatic), 7.31 (*d*, *J* = 8 Hz, 4H, C*H* aromatic), 7.20 (*s*, 2H, C*H* imidazole), 2.48 [*sept*, *J* = 7 Hz, 4H, C*H*(CH_3_)_2_], 1.30 [*d*, *J* = 7 Hz, 12H, CH(C*H*_3_)_2_], 1.21 [*d*, *J* = 7 Hz, 12H, CH(C*H*_3_)_2_]. ^13^C NMR (100 MHz, CDCl_3_): *δ* 183.4 (*s*, *C* carbene), 144.1 (*s*, *C* aromatic), 133.8 (*s*, *C* aromatic), 132.3 (*s*, N*C*O), 131.2 (*s*, CH imidazole), 123.9 (*s*, *C*H aromatic), 123.4 (*s*, *C*H aromatic), 29.8 [*s*, *C*H(CH_3_)_2_], 25.5 [*s*, CH(*C*H_3_)_2_], 24.0 [*s*, CH(*C*H_3_)_2_]. Analysis calculated for C_28_H_36_AuN_3_O: C, 53.59; H, 5.78; N, 6.70. Found: C, 53.58; H, 5.82; N, 6.52.

## 6. Refinement

Crystal data, data collection and structure refinement details are summarized in Table 1 ▸. Atoms H2 and H3 were refined in an isotropic approximation. Other H atoms were treated as riding in idealized positions (for methyl groups, optimized by rotation about *R*—CH_3_ bonds) with *U*_iso_(H) = 1.5*U*_eq_ for methyl H atoms, or 1.2*U*_eq_(C) for the rest.

## Supplementary Material

Crystal structure: contains datablock(s) I. DOI: 10.1107/S205698902400046X/zv2031sup1.cif

Structure factors: contains datablock(s) I. DOI: 10.1107/S205698902400046X/zv2031Isup5.hkl

CCDC reference: 2306677

Additional supporting information:  crystallographic information; 3D view; checkCIF report

## Figures and Tables

**Table 1:** Experimental details

Crystal data	
Chemical formula	[Au(NCO)(C_27_H_36_N_2_)]
*M*_r_	627.56
Crystal system, space group	Orthorhombic, *P*2_1_2_1_2_1_
Temperature (K)	100
*a*, *b*, *c* (Å)	10.3941 (7), 11.1540 (7), 23.3489 (15)
*V* (Å^3^)	2707.0 (3)
*Z*	4
Radiation type	Mo *K*α
μ (mm^−1^)	5.46
Crystal size (mm)	0.18 × 0.17 × 0.12

Data collection	
Diffractometer	Bruker D8 Quest/Photon 100
Absorption correction	Multi-scan (*SADABS*; Krause *et al.*, 2015 ▸)
*T*_min_, *T*_max_	0.512, 0.710
No. of measured, independent and observed [*I* > 2σ(*I*)] reflections	76237, 6680, 6610
*R*_int_	0.029
(sin θ/λ)_max_ (Å^−1^)	0.668

Refinement	
*R*[*F* ^2^ > 2σ(*F* ^2^)], *wR*(*F* ^2^), *S*	0.013, 0.025, 1.11
No. of reflections	6680
No. of parameters	315
H-atom treatment	H atoms treated by a mixture of independent and constrained refinement
Δρ_max_, Δρ_min_ (e Å^−3^)	0.61, −0.31
Absolute structure	Flack *x* determined using 2845 quotients [(*I* ^+^)−(*I* ^−^)]/[(*I* ^+^)+(*I* ^−^)] (Parsons *et al.*, 2013 ▸)
Absolute structure parameter	0.007 (2)

Computer programs: *APEX3*, *SAINT*, *XPREP* and *XCIF* (Bruker, 2016 ▸), *SHELXT2014/5* (Sheldrick, 2015*a* ▸), *SHELXL2019/1* (Sheldrick, 2015*b* ▸) and *ShelXle* v932 (Hübschle *et al.*, 2011 ▸).

## supplementary crystallographic information

### Crystal data

**Table:**

[Au(NCO)(C_27_H_36_N_2_)]	*D*_x_ = 1.540 Mg m^−^^3^
*M**_r_* = 627.56	Mo *K*α radiation, λ = 0.71073 Å
Orthorhombic, *P*2_1_2_1_2_1_	Cell parameters from 9267 reflections
*a* = 10.3941 (7) Å	θ = 2.6–28.3°
*b* = 11.1540 (7) Å	µ = 5.46 mm^−^^1^
*c* = 23.3489 (15) Å	*T* = 100 K
*V* = 2707.0 (3) Å^3^	Prism, colourless
*Z* = 4	0.18 × 0.17 × 0.12 mm
*F*(000) = 1248	

### Data collection

**Table:**

Bruker D8 Quest/Photon 100 diffractometer	6680 independent reflections
Radiation source: microfocus sealed tube	6610 reflections with *I* > 2σ(*I*)
Multilayer mirrors monochromator	*R*_int_ = 0.029
profile data from φ and ω scans	θ_max_ = 28.4°, θ_min_ = 2.5°
Absorption correction: multi-scan (SADABS; Krause *et al.*, 2015)	*h* = −13→13
*T*_min_ = 0.512, *T*_max_ = 0.710	*k* = −14→14
76237 measured reflections	*l* = −31→31

### Refinement

**Table:**

Refinement on *F*^2^	Hydrogen site location: inferred from neighbouring sites
Least-squares matrix: full	H atoms treated by a mixture of independent and constrained refinement
*R*[*F*^2^ > 2σ(*F*^2^)] = 0.013	*w* = 1/[σ^2^(*F*_o_^2^) + (0.0022*P*)^2^ + 0.5084*P*] where *P* = (*F*_o_^2^ + 2*F*_c_^2^)/3
*wR*(*F*^2^) = 0.025	(Δ/σ)_max_ = 0.003
*S* = 1.11	Δρ_max_ = 0.61 e Å^−^^3^
6680 reflections	Δρ_min_ = −0.31 e Å^−^^3^
315 parameters	Extinction correction: *SHELXL2019/1* (Sheldrick, 2015b), Fc^*^=kFc[1+0.001xFc^2^λ^3^/sin(2θ)]^-1/4^
0 restraints	Extinction coefficient: 0.00303 (9)
Primary atom site location: dual	Absolute structure: Flack *x* determined using 2845 quotients [(*I*^+^)-(*I*^-^)]/[(*I*^+^)+(*I*^-^)] (Parsons *et al*., 2013)
Secondary atom site location: difference Fourier map	Absolute structure parameter: 0.007 (2)

### Special details

**Table:**

Experimental. One distinct cell was identified using APEX3 (Bruker, 2016). Six frame series were integrated and filtered for statistical outliers using SAINT (Bruker, 2016) then corrected for absorption by integration using SAINT/SADABS v2014/2 (Bruker, 2016) to sort, merge, and scale the combined data. No decay correction was applied.
Geometry. All esds (except the esd in the dihedral angle between two l.s. planes) are estimated using the full covariance matrix. The cell esds are taken into account individually in the estimation of esds in distances, angles and torsion angles; correlations between esds in cell parameters are only used when they are defined by crystal symmetry. An approximate (isotropic) treatment of cell esds is used for estimating esds involving l.s. planes.
Refinement. Structure was phased by intrinsic methods (Sheldrick, 2015a). Systematic conditions suggested the ambiguous space group. The space group choice was confirmed by successful convergence of the full-matrix least-squares refinement on F^2^. The final map had no significant features. A final analysis of variance between observed and calculated structure factors showed little dependence on amplitude and resolution.

### Fractional atomic coordinates and isotropic or equivalent isotropic displacement parameters (Å^2^)

**Table:**

	*x*	*y*	*z*	*U*_iso_*/*U*_eq_	
Au1	0.26753 (2)	0.23607 (2)	0.37741 (2)	0.01629 (3)	
O1	0.2821 (2)	−0.14249 (15)	0.41879 (9)	0.0378 (5)	
N1	0.2343 (2)	0.49912 (15)	0.39199 (7)	0.0157 (4)	
N2	0.2784 (2)	0.45853 (16)	0.30454 (7)	0.0151 (4)	
N3	0.2667 (3)	0.06303 (19)	0.39972 (9)	0.0283 (5)	
C1	0.2625 (2)	0.40636 (18)	0.35634 (9)	0.0148 (4)	
C2	0.2332 (2)	0.6070 (2)	0.36284 (10)	0.0197 (5)	
H2	0.221 (2)	0.680 (2)	0.3819 (10)	0.023 (7)*	
C3	0.2606 (3)	0.58160 (19)	0.30804 (10)	0.0190 (5)	
H3	0.270 (3)	0.629 (2)	0.2773 (10)	0.017 (6)*	
C4	0.2132 (2)	0.4847 (2)	0.45287 (9)	0.0168 (5)	
C5	0.0907 (2)	0.4478 (2)	0.47107 (11)	0.0193 (5)	
C6	0.0742 (3)	0.4320 (2)	0.52988 (12)	0.0246 (6)	
H6	−0.006924	0.406579	0.544201	0.029*	
C7	0.1742 (3)	0.4528 (2)	0.56757 (11)	0.0267 (6)	
H7	0.161106	0.441254	0.607458	0.032*	
C8	0.2930 (3)	0.4900 (2)	0.54798 (10)	0.0259 (6)	
H8	0.360466	0.503938	0.574613	0.031*	
C9	0.3155 (2)	0.5077 (2)	0.48950 (10)	0.0208 (5)	
C10	−0.0199 (2)	0.4307 (2)	0.42925 (12)	0.0229 (6)	
H10	0.017665	0.416710	0.390388	0.027*	
C11	−0.1042 (3)	0.3230 (3)	0.44407 (15)	0.0394 (8)	
H11A	−0.171818	0.314256	0.415116	0.059*	
H11B	−0.143619	0.335255	0.481746	0.059*	
H11C	−0.051310	0.250252	0.444865	0.059*	
C12	−0.1010 (3)	0.5452 (3)	0.42656 (13)	0.0329 (7)	
H12A	−0.046152	0.612932	0.415753	0.049*	
H12B	−0.139480	0.560496	0.464170	0.049*	
H12C	−0.169343	0.535288	0.398019	0.049*	
C13	0.4476 (3)	0.5458 (3)	0.46822 (11)	0.0264 (6)	
H13	0.438282	0.567922	0.426931	0.032*	
C14	0.4986 (3)	0.6556 (3)	0.49942 (12)	0.0413 (8)	
H14A	0.581927	0.678645	0.483126	0.062*	
H14B	0.509191	0.637001	0.540165	0.062*	
H14C	0.437623	0.721984	0.495059	0.062*	
C15	0.5423 (3)	0.4419 (3)	0.47129 (18)	0.0453 (9)	
H15A	0.504052	0.370822	0.453394	0.068*	
H15B	0.562278	0.424411	0.511450	0.068*	
H15C	0.621502	0.463457	0.450996	0.068*	
C16	0.3151 (2)	0.3971 (2)	0.25267 (10)	0.0171 (5)	
C17	0.4467 (3)	0.3911 (2)	0.23968 (11)	0.0207 (6)	
C18	0.4805 (3)	0.3365 (2)	0.18834 (11)	0.0270 (6)	
H18	0.568715	0.330687	0.177981	0.032*	
C19	0.3877 (3)	0.2906 (3)	0.15223 (11)	0.0293 (6)	
H19	0.412772	0.254023	0.117211	0.035*	
C20	0.2586 (3)	0.2971 (2)	0.16641 (10)	0.0266 (5)	
H20	0.196098	0.264555	0.141130	0.032*	
C21	0.2193 (3)	0.3511 (2)	0.21743 (10)	0.0205 (5)	
C22	0.5492 (3)	0.4415 (3)	0.27965 (12)	0.0259 (6)	
H22	0.503778	0.484144	0.311436	0.031*	
C23	0.6350 (4)	0.5327 (4)	0.24968 (15)	0.0524 (10)	
H23A	0.681140	0.493551	0.218227	0.079*	
H23B	0.697090	0.565354	0.277140	0.079*	
H23C	0.581895	0.597885	0.234403	0.079*	
C24	0.6274 (4)	0.3407 (3)	0.30637 (17)	0.0595 (11)	
H24A	0.679597	0.301835	0.276762	0.089*	
H24B	0.569013	0.281642	0.323466	0.089*	
H24C	0.683788	0.373548	0.336097	0.089*	
C25	0.0776 (3)	0.3555 (3)	0.23361 (12)	0.0261 (6)	
H25	0.068802	0.410186	0.267309	0.031*	
C26	0.0316 (3)	0.2308 (3)	0.25208 (13)	0.0390 (7)	
H26A	0.036322	0.175785	0.219407	0.059*	
H26B	−0.057475	0.235788	0.265587	0.059*	
H26C	0.086646	0.200985	0.283054	0.059*	
C27	−0.0068 (3)	0.4051 (3)	0.18560 (13)	0.0375 (8)	
H27A	0.025228	0.484162	0.174054	0.056*	
H27B	−0.095587	0.412489	0.199211	0.056*	
H27C	−0.004031	0.350569	0.152718	0.056*	
C28	0.2743 (3)	−0.0363 (2)	0.40865 (10)	0.0218 (5)	

### Atomic displacement parameters (Å^2^)

**Table:**

	*U*^11^	*U*^22^	*U*^33^	*U*^12^	*U*^13^	*U*^23^
Au1	0.01867 (5)	0.01490 (5)	0.01529 (4)	0.00074 (3)	−0.00236 (3)	0.00049 (3)
O1	0.0479 (13)	0.0154 (9)	0.0502 (12)	−0.0026 (9)	0.0055 (11)	0.0016 (8)
N1	0.0162 (10)	0.0178 (10)	0.0132 (9)	0.0007 (8)	−0.0020 (8)	−0.0020 (6)
N2	0.0162 (10)	0.0153 (9)	0.0137 (9)	0.0017 (9)	−0.0004 (8)	−0.0009 (7)
N3	0.0358 (14)	0.0224 (11)	0.0268 (11)	0.0023 (12)	−0.0081 (11)	0.0032 (8)
C1	0.0122 (11)	0.0174 (10)	0.0148 (10)	−0.0004 (10)	−0.0013 (9)	0.0008 (8)
C2	0.0213 (12)	0.0143 (10)	0.0235 (12)	0.0016 (10)	−0.0008 (11)	−0.0022 (9)
C3	0.0213 (13)	0.0164 (11)	0.0192 (11)	0.0002 (11)	−0.0006 (11)	0.0034 (9)
C4	0.0198 (12)	0.0179 (11)	0.0125 (10)	0.0034 (10)	0.0006 (9)	−0.0020 (8)
C5	0.0163 (12)	0.0209 (13)	0.0208 (13)	0.0043 (10)	0.0011 (10)	−0.0030 (10)
C6	0.0224 (15)	0.0298 (15)	0.0215 (14)	0.0038 (11)	0.0067 (11)	−0.0012 (11)
C7	0.0326 (15)	0.0326 (16)	0.0149 (12)	0.0032 (12)	0.0035 (11)	−0.0016 (11)
C8	0.0252 (14)	0.0341 (15)	0.0184 (12)	0.0012 (11)	−0.0049 (10)	−0.0032 (10)
C9	0.0204 (12)	0.0225 (14)	0.0194 (13)	−0.0002 (10)	−0.0014 (10)	−0.0016 (10)
C10	0.0155 (12)	0.0300 (15)	0.0232 (14)	0.0001 (11)	0.0009 (10)	−0.0024 (11)
C11	0.0345 (17)	0.0296 (16)	0.054 (2)	−0.0079 (13)	−0.0191 (15)	0.0070 (14)
C12	0.0256 (15)	0.0304 (16)	0.0425 (17)	0.0015 (12)	−0.0100 (13)	0.0063 (13)
C13	0.0205 (13)	0.0397 (17)	0.0190 (13)	−0.0062 (12)	−0.0051 (11)	0.0025 (12)
C14	0.046 (2)	0.0443 (19)	0.0339 (19)	−0.0176 (16)	−0.0041 (15)	0.0028 (14)
C15	0.0194 (17)	0.048 (2)	0.068 (3)	−0.0007 (15)	0.0118 (16)	0.0029 (18)
C16	0.0225 (12)	0.0167 (11)	0.0121 (11)	0.0022 (10)	0.0013 (9)	0.0012 (9)
C17	0.0229 (13)	0.0197 (13)	0.0194 (13)	0.0014 (11)	0.0024 (10)	0.0015 (10)
C18	0.0259 (15)	0.0301 (15)	0.0249 (14)	0.0052 (12)	0.0070 (11)	0.0020 (12)
C19	0.0391 (16)	0.0325 (16)	0.0163 (12)	0.0090 (12)	0.0028 (11)	−0.0049 (11)
C20	0.0339 (15)	0.0279 (12)	0.0180 (11)	0.0044 (12)	−0.0064 (11)	−0.0050 (9)
C21	0.0233 (13)	0.0205 (11)	0.0176 (11)	0.0015 (11)	−0.0019 (10)	0.0014 (9)
C22	0.0192 (14)	0.0299 (16)	0.0287 (15)	−0.0010 (12)	0.0051 (11)	−0.0059 (12)
C23	0.047 (2)	0.059 (2)	0.051 (2)	−0.027 (2)	0.0122 (18)	−0.0087 (19)
C24	0.060 (2)	0.047 (2)	0.071 (3)	0.0126 (18)	−0.045 (2)	−0.0157 (19)
C25	0.0215 (14)	0.0371 (17)	0.0198 (14)	0.0017 (12)	−0.0026 (11)	−0.0029 (12)
C26	0.0282 (14)	0.0491 (19)	0.0397 (16)	−0.0129 (15)	−0.0086 (12)	0.0063 (16)
C27	0.0280 (16)	0.054 (2)	0.0302 (17)	0.0101 (15)	−0.0068 (13)	−0.0018 (15)
C28	0.0220 (13)	0.0270 (14)	0.0163 (12)	−0.0029 (12)	0.0008 (11)	−0.0052 (9)

### Geometric parameters (Å, º)

**Table:**

Au1—N3	1.999 (2)	C14—H14A	0.9800
Au1—C1	1.963 (2)	C14—H14B	0.9800
O1—C28	1.210 (3)	C14—H14C	0.9800
N1—C1	1.360 (3)	C15—H15A	0.9800
N1—C2	1.382 (3)	C15—H15B	0.9800
N1—C4	1.447 (3)	C15—H15C	0.9800
N2—C1	1.352 (3)	C16—C17	1.402 (4)
N2—C3	1.387 (3)	C16—C21	1.390 (3)
N2—C16	1.443 (3)	C17—C18	1.390 (4)
N3—C28	1.130 (3)	C17—C22	1.524 (4)
C2—H2	0.94 (3)	C18—H18	0.9500
C2—C3	1.341 (3)	C18—C19	1.380 (4)
C3—H3	0.89 (2)	C19—H19	0.9500
C4—C5	1.404 (3)	C19—C20	1.384 (4)
C4—C9	1.388 (3)	C20—H20	0.9500
C5—C6	1.395 (4)	C20—C21	1.396 (3)
C5—C10	1.520 (3)	C21—C25	1.521 (4)
C6—H6	0.9500	C22—H22	1.0000
C6—C7	1.382 (4)	C22—C23	1.523 (4)
C7—H7	0.9500	C22—C24	1.521 (4)
C7—C8	1.381 (4)	C23—H23A	0.9800
C8—H8	0.9500	C23—H23B	0.9800
C8—C9	1.399 (3)	C23—H23C	0.9800
C9—C13	1.521 (4)	C24—H24A	0.9800
C10—H10	1.0000	C24—H24B	0.9800
C10—C11	1.527 (4)	C24—H24C	0.9800
C10—C12	1.532 (4)	C25—H25	1.0000
C11—H11A	0.9800	C25—C26	1.533 (4)
C11—H11B	0.9800	C25—C27	1.527 (4)
C11—H11C	0.9800	C26—H26A	0.9800
C12—H12A	0.9800	C26—H26B	0.9800
C12—H12B	0.9800	C26—H26C	0.9800
C12—H12C	0.9800	C27—H27A	0.9800
C13—H13	1.0000	C27—H27B	0.9800
C13—C14	1.521 (4)	C27—H27C	0.9800
C13—C15	1.522 (4)		
			
C1—Au1—N3	178.14 (11)	H14A—C14—H14C	109.5
C1—N1—C2	111.26 (18)	H14B—C14—H14C	109.5
C1—N1—C4	123.35 (18)	C13—C15—H15A	109.5
C2—N1—C4	125.36 (18)	C13—C15—H15B	109.5
C1—N2—C3	110.91 (18)	C13—C15—H15C	109.5
C1—N2—C16	125.33 (18)	H15A—C15—H15B	109.5
C3—N2—C16	123.69 (18)	H15A—C15—H15C	109.5
C28—N3—Au1	173.8 (2)	H15B—C15—H15C	109.5
N1—C1—Au1	126.05 (16)	C17—C16—N2	117.5 (2)
N2—C1—Au1	129.61 (16)	C21—C16—N2	118.8 (2)
N2—C1—N1	104.26 (17)	C21—C16—C17	123.6 (2)
N1—C2—H2	121.6 (15)	C16—C17—C22	122.1 (2)
C3—C2—N1	106.53 (19)	C18—C17—C16	117.0 (2)
C3—C2—H2	131.7 (15)	C18—C17—C22	120.8 (2)
N2—C3—H3	121.3 (15)	C17—C18—H18	119.6
C2—C3—N2	107.05 (19)	C19—C18—C17	120.8 (3)
C2—C3—H3	131.7 (15)	C19—C18—H18	119.6
C5—C4—N1	117.9 (2)	C18—C19—H19	119.6
C9—C4—N1	117.9 (2)	C18—C19—C20	120.8 (2)
C9—C4—C5	124.2 (2)	C20—C19—H19	119.6
C4—C5—C10	121.9 (2)	C19—C20—H20	119.6
C6—C5—C4	116.5 (2)	C19—C20—C21	120.7 (2)
C6—C5—C10	121.5 (2)	C21—C20—H20	119.6
C5—C6—H6	119.6	C16—C21—C20	117.0 (2)
C7—C6—C5	120.9 (3)	C16—C21—C25	122.3 (2)
C7—C6—H6	119.6	C20—C21—C25	120.6 (2)
C6—C7—H7	119.6	C17—C22—H22	107.4
C8—C7—C6	120.8 (2)	C23—C22—C17	112.0 (2)
C8—C7—H7	119.6	C23—C22—H22	107.4
C7—C8—H8	119.5	C24—C22—C17	110.6 (2)
C7—C8—C9	121.0 (2)	C24—C22—H22	107.4
C9—C8—H8	119.5	C24—C22—C23	111.7 (3)
C4—C9—C8	116.6 (2)	C22—C23—H23A	109.5
C4—C9—C13	122.8 (2)	C22—C23—H23B	109.5
C8—C9—C13	120.6 (2)	C22—C23—H23C	109.5
C5—C10—H10	107.9	H23A—C23—H23B	109.5
C5—C10—C11	112.8 (2)	H23A—C23—H23C	109.5
C5—C10—C12	109.8 (2)	H23B—C23—H23C	109.5
C11—C10—H10	107.9	C22—C24—H24A	109.5
C11—C10—C12	110.4 (2)	C22—C24—H24B	109.5
C12—C10—H10	107.9	C22—C24—H24C	109.5
C10—C11—H11A	109.5	H24A—C24—H24B	109.5
C10—C11—H11B	109.5	H24A—C24—H24C	109.5
C10—C11—H11C	109.5	H24B—C24—H24C	109.5
H11A—C11—H11B	109.5	C21—C25—H25	107.7
H11A—C11—H11C	109.5	C21—C25—C26	110.0 (2)
H11B—C11—H11C	109.5	C21—C25—C27	112.7 (2)
C10—C12—H12A	109.5	C26—C25—H25	107.7
C10—C12—H12B	109.5	C27—C25—H25	107.7
C10—C12—H12C	109.5	C27—C25—C26	110.9 (2)
H12A—C12—H12B	109.5	C25—C26—H26A	109.5
H12A—C12—H12C	109.5	C25—C26—H26B	109.5
H12B—C12—H12C	109.5	C25—C26—H26C	109.5
C9—C13—H13	107.2	H26A—C26—H26B	109.5
C9—C13—C15	110.8 (2)	H26A—C26—H26C	109.5
C14—C13—C9	112.6 (2)	H26B—C26—H26C	109.5
C14—C13—H13	107.2	C25—C27—H27A	109.5
C14—C13—C15	111.4 (3)	C25—C27—H27B	109.5
C15—C13—H13	107.2	C25—C27—H27C	109.5
C13—C14—H14A	109.5	H27A—C27—H27B	109.5
C13—C14—H14B	109.5	H27A—C27—H27C	109.5
C13—C14—H14C	109.5	H27B—C27—H27C	109.5
H14A—C14—H14B	109.5	N3—C28—O1	179.3 (3)
			
N1—C2—C3—N2	−0.1 (3)	C5—C6—C7—C8	0.2 (4)
N1—C4—C5—C6	178.5 (2)	C6—C5—C10—C11	−40.7 (4)
N1—C4—C5—C10	−3.9 (3)	C6—C5—C10—C12	82.9 (3)
N1—C4—C9—C8	−178.4 (2)	C6—C7—C8—C9	−0.1 (4)
N1—C4—C9—C13	−0.5 (4)	C7—C8—C9—C4	−0.5 (4)
N2—C16—C17—C18	−177.2 (2)	C7—C8—C9—C13	−178.5 (3)
N2—C16—C17—C22	3.0 (4)	C8—C9—C13—C14	−51.9 (3)
N2—C16—C21—C20	177.1 (2)	C8—C9—C13—C15	73.7 (3)
N2—C16—C21—C25	−4.4 (3)	C9—C4—C5—C6	−1.0 (4)
C1—N1—C2—C3	0.2 (3)	C9—C4—C5—C10	176.5 (2)
C1—N1—C4—C5	−82.3 (3)	C10—C5—C6—C7	−177.2 (2)
C1—N1—C4—C9	97.3 (3)	C16—N2—C1—Au1	−6.1 (4)
C1—N2—C3—C2	0.0 (3)	C16—N2—C1—N1	177.3 (2)
C1—N2—C16—C17	−91.9 (3)	C16—N2—C3—C2	−177.2 (2)
C1—N2—C16—C21	90.3 (3)	C16—C17—C18—C19	0.0 (4)
C2—N1—C1—Au1	−177.09 (17)	C16—C17—C22—C23	−124.0 (3)
C2—N1—C1—N2	−0.3 (3)	C16—C17—C22—C24	110.7 (3)
C2—N1—C4—C5	100.1 (3)	C16—C21—C25—C26	−105.2 (3)
C2—N1—C4—C9	−80.3 (3)	C16—C21—C25—C27	130.5 (3)
C3—N2—C1—Au1	176.85 (18)	C17—C16—C21—C20	−0.4 (4)
C3—N2—C1—N1	0.2 (3)	C17—C16—C21—C25	178.1 (2)
C3—N2—C16—C17	84.8 (3)	C17—C18—C19—C20	−0.4 (4)
C3—N2—C16—C21	−92.9 (3)	C18—C17—C22—C23	56.1 (4)
C4—N1—C1—Au1	5.0 (4)	C18—C17—C22—C24	−69.2 (4)
C4—N1—C1—N2	−178.2 (2)	C18—C19—C20—C21	0.4 (4)
C4—N1—C2—C3	178.1 (2)	C19—C20—C21—C16	0.0 (4)
C4—C5—C6—C7	0.3 (4)	C19—C20—C21—C25	−178.5 (2)
C4—C5—C10—C11	141.9 (3)	C20—C21—C25—C26	73.2 (3)
C4—C5—C10—C12	−94.5 (3)	C20—C21—C25—C27	−51.1 (3)
C4—C9—C13—C14	130.3 (3)	C21—C16—C17—C18	0.4 (4)
C4—C9—C13—C15	−104.1 (3)	C21—C16—C17—C22	−179.4 (2)
C5—C4—C9—C8	1.1 (4)	C22—C17—C18—C19	179.8 (3)
C5—C4—C9—C13	179.0 (2)		

### Hydrogen-bond geometry (Å, º)

**Table:**

*D*—H···*A*	*D*—H	H···*A*	*D*···*A*	*D*—H···*A*
C2—H2···O1^i^	0.94 (3)	2.25 (3)	3.127 (3)	155 (2)

Symmetry code: (i) *x*, *y*+1, *z*.
